# Proteomic profiling of NCI-60 extracellular vesicles uncovers common protein cargo and cancer type-specific biomarkers

**DOI:** 10.18632/oncotarget.13569

**Published:** 2016-11-24

**Authors:** Stephanie N. Hurwitz, Mark A. Rider, Joseph L. Bundy, Xia Liu, Rakesh K. Singh, David G. Meckes

**Affiliations:** ^1^ Department of Biomedical Sciences, Florida State University College of Medicine, Tallahassee, FL, 32306, USA

**Keywords:** exosomes, microvesicles, co-inertia, proteomics, biomarkers

## Abstract

Packed with biological information, extracellular vesicles (EVs) offer exciting promise for biomarker discovery and applications in therapeutics and non-invasive diagnostics. Currently, our understanding of EV contents is confined by the limited cells from which vesicles have been characterized utilizing the same enrichment method. Using sixty cell lines from the National Cancer Institute (NCI-60), here we provide the largest proteomic profile of EVs in a single study, identifying 6,071 proteins with 213 common to all isolates. Proteins included established EV markers, and vesicular trafficking proteins such as Rab GTPases and tetraspanins. Differentially-expressed proteins offer potential for cancer diagnosis and prognosis. Network analysis of vesicle quantity and proteomes identified EV components associated with vesicle secretion, including CD81, CD63, syntenin-1, VAMP3, Rab GTPases, and integrins. Integration of vesicle proteomes with whole-cell molecular profiles revealed similarities, suggesting EVs provide a reliable reflection of their progenitor cell content, and are therefore excellent indicators of disease.

## INTRODUCTION

Extracellular vesicles (EVs) represent a diverse population of communication pods released from cells. Ranging in size from 40 to 1000 nm, EVs include exosomes, microvesicles, and apoptotic bodies. Generally, exosomes are described as 40-150 nm endocytically-derived vesicles formed by intraluminal budding of multivesicular bodies (MVBs) which are released following MVB fusion with the plasma membrane. Microvesicles are generally larger than exosomes, and are shed by budding and fission events directly at the cell membrane. Varying in size, apoptotic bodies are formed by plasma membrane blebbing, and can contain packaged organelles following initiation of cell death. Further sub-populations of vesicles likely exist, reflecting the heterogeneity of cellular biology encapsulated by EVs.

Extracellular vesicles have been implicated in a number of different physiological processes, including immune system modulation, cell-to-cell signaling, and cell proliferation [1–5]. Accumulating evidence has implicated EVs as major players in the growth, invasion, and metastatic capacity of cancer cells [6, 7]. For example, exosomes and microvesicles have been demonstrated to transfer oncoproteins and nucleic acids from virally-infected cells to uninfected neighboring cells, and likely promote viral-associated tumor progression [8–11]. Systemic circulation of EVs can play a role in establishing a tumor microenvironment, providing the “soil” for cancer cell “seeding” to metastatic sites [12] and cancer patients have been shown to have increased levels of circulating EVs [13–15]. Recent research has begun to characterize specific transmembrane proteins responsible for targeted vesicle uptake by cells in common cancer type-specific metastatic sites [16]. These circulating vesicles therefore reflect a diverse form of intercellular communication that can facilitate the progression of neoplastic growth and tumor metastasis.

Though EVs likely contribute to the progression of some types of cancer, our knowledge of vesicular communication is still incomplete. One reason is due to the heterogeneity of EV sub-populations. Though the complexity of EV populations has burdened our understanding of their roles in cell biology, the existence of a variety of EVs may be beneficial in the context of cancer diagnostics and prognostics. One challenge in diagnosing cancer is that tumors often represent a diversity of cell types and genetic mutations; a tissue biopsy is limited in its ability to reflect this diversity. However, circulating EVs are derived from an all-inclusive population of cells, and therefore have the potential to more accurately reflect the entirety of a heterogeneous tumor [17]. While some limitations of vesicle-based biopsies exist, such as disparities in EV quantities released from different tumor cells and the ability to detect small changes in the populations of circulating vesicles, EV-based detection offers alternatives to current diagnostic approaches. Current screening or monitoring tests of cancer progression, such as prostate-specific antigen (PSA; prostate), CA-125 (ovarian), alpha fetoprotein (liver), or CA19-9 (pancreatic) often lack the sensitivity or specificity to provide highly accurate clinical diagnoses [18–20]. As EVs provide membrane-bound protected cargo that can reflect cell-specific pathological processes, we and others propose that these vesicles bear great potential as circulating biomarkers that could improve the current strategies of cancer diagnosis [21–24].

A better understanding of the contents of these vesicles is crucial to the development of EV clinical applications. A current limitation in proteomic analyses of EVs from cancer cells is the narrow number of cell lines studied using comparable and reproducible methods. Here, we characterize 60 diverse human cancer cells derived from 9 distinct tissue types from the National Cancer Institute (NCI-60). The NCI-60 panel was originally compiled by the Developmental Therapeutics Program for high-throughput drug screening, and has led to a number of successful chemotherapeutic drugs used to treat cancer patients [25]. The NCI-60 has also contributed vastly to a better understanding of cancer cell biology and the identification of many novel oncogenic DNA mutations [26, 27]. Since then, the panel has become publicized for cancer research purposes, and a full whole-cell proteomic analysis of each individual cell line has been published [28]. Proteomic and RNA analyses of EVs from subsets of the NCI-60 panel have recently been investigated, providing initial characterizations of cancer vesicle contents [29–32]. Research using cell lines from the NCI-60 panel has also contributed to evidence demonstrating the roles of EVs in the growth and survival of tumor cells, multidrug resistance [33–35], immune evasion [36], cancer cell migration [37] and impact on cells in the tumor microenvironment [38]. Subsets of the panel have also been used to study general mechanisms of EV biogenesis and release from cells [39, 40]. Recently, we compared the vesicle secretion of NCI-60 cell lines using nanoparticle tracking analysis (NTA). Results highlighted differences in secretion rates and sizes of vesicles from cancer cells [41].

With this in mind, we conducted a comparative analysis of proteins from EVs secreted by the NCI-60 cells. To our knowledge, this is the largest single-study proteomic investigation of vesicles to date. In this study, 6,071 unique proteins were identified, including 213 common to all 60 cell types, which likely reflect the common machinery involved in EV biogenesis. Differentially expressed proteins were also identified. Many of these proteins are associated with tissue type, and could therefore serve as markers of EV origin or aid as future diagnostic biomarkers of cancers. To investigate proteins involved in mechanisms of EV biogenesis and secretion, the EV proteome was further analyzed to look for associations between protein accumulation (spectral counts) and vesicle secretion quantity. Finally, the proteomic analysis of NCI-60 EVs was compared to existing cellular proteome and transcriptome datasets. These analyses revealed that the EV proteome closely reflects the transcriptome and proteome of the cell of origin, supporting the hypothesis that EVs are a rich source of diagnostic and prognostic markers. Overall, this extensive proteomic dataset provides a foundation to further investigate general mechanisms of vesicle biogenesis, and demonstrates the incredible biomedical and clinical utilities of extracellular vesicles.

## RESULTS AND DISCUSSION

### Cancer-cell derived EVs contain core vesicle machinery

**Figure 1:**
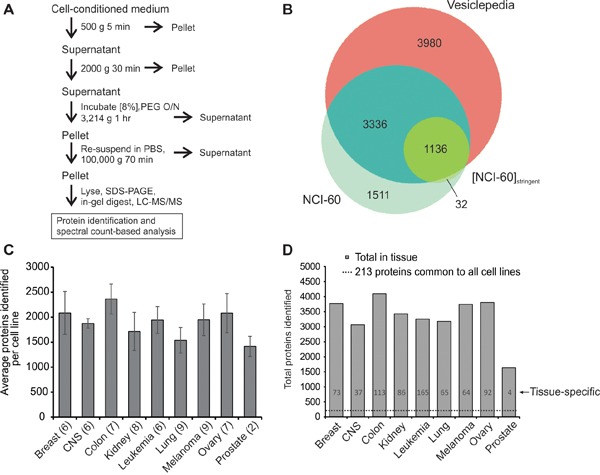
Proteomic analysis of extracellular vesicles secreted by the NCI-60 cells **A.** Centrifugation protocol and general workflow of EV enrichment for LC-MS/MS analysis. **B.** Venn diagram of proteins identified in EV samples in the NCI-60 and NCI-60]_stringent_ datasets compared to the Vesiclepedia database of proteins. See also Supplementary Table S2. **C.** Average spectral counts per cell line across tissue types. Parentheses indicate the number of cell lines represented in each tissue type. Data are represented as mean ± STD. **D.** Total proteins including common proteome (dotted line) and tissue-specific proteins identified across the nine histological origins represented. See also Supplementary Table S3 and S4.

To characterize and compare extracellular vesicle proteomes, EVs were harvested from 60 cell lines (NCI-60). As pure EV sub-populations are empirically difficult to isolate, a method of enriching a broad spectrum of vesicles was used in this study to contribute to a greater understanding of global EV content. We have previously demonstrated that the ExtraPEG method enriches for EVs with a comparable purity [42] to sucrose-purified samples following growth of cells for a period of time in serum-free medium, as performed in this study [43] (Figure 1A). Vesicles harvested using this method were found to contain extracellular vesicle markers by western blot [41, 43]. Nanoparticle tracking and electron microscopy revealed sizes and morphology consistent with those previously reported for EVs [41, 43]. Here, vesicles were enriched using identical methods from individual cell lines across nine represented histological origins: breast, brain (CNS), colon, kidney, leukemia, lung, melanoma, ovary, and prostate. Collectively, 6,071 unique proteins were identified in EVs (Supplementary Table S1). To examine the overlap between known vesicular proteins, NCI-60 EV proteins were compared to those in the Vesiclepedia compendium of extracellular vesicle molecular data [44]. Nearly 4,500 proteins were previously identified in EVs (Figure 1B and Supplementary Table S2). Over 1,500 proteins not previously characterized as EV components were further discovered. Because so many unreported vesicular proteins were identified, we aimed to ensure that proteins found in this study were congruent with those previously found in EVs. To increase the stringency of our dataset and characterize common EV proteins, only those identified in at least two-thirds all cell line isolates ([NCI-60]_stringent_) were compared to the Vesiclepedia compendium. These proteins showed over 97% overlap with proteins currently characterized as extracellular vesicle proteins.

The entire proteome of NCI-60-derived EVs was further characterized systematically using qualitative and quantitative analyses. An average of nearly 1,900 proteins were identified per cell line across all tissue types (Figure 1C). Calculation of the median logarithmic abundance of proteins revealed a normal distribution of spectral counts across the panel (Supplementary Figure 1D). The number of total EV proteins found within each tissue was similar across the panel (Figure 1D), with the exception of the prostate cancer group, likely explained by the underrepresentation of this tissue type (n=2). Strikingly, 213 proteins were identified that were common to every vesicle sample, representing the core NCI-60 EV proteome (Supplementary Table S3). These proteins likely reflect essential proteins packaged into EVs from many different cellular origins, and provide insight into general mechanisms of EV biogenesis, entry, protein trafficking, and secretion. Proteins found in at least one cell line from each exclusive tissue type were also compiled to represent tissue-specific markers (Supplementary Table S4). Notably, 165 proteins were exclusively found in leukemia-derived EVs, while fewer unique proteins were in other tissue types. As EVs from many cancer cells have been shown to be enriched in functional integrins [16, 45], these data may reflect detectable differences between cancer EVs originating from circulating hematopoietic cells versus those typically attached to basement membrane matrices through integrin linkers.

### Enrichment analysis highlights the subcellular localization and function of cancer cell extracellular vesicle proteins

**Figure 2:**
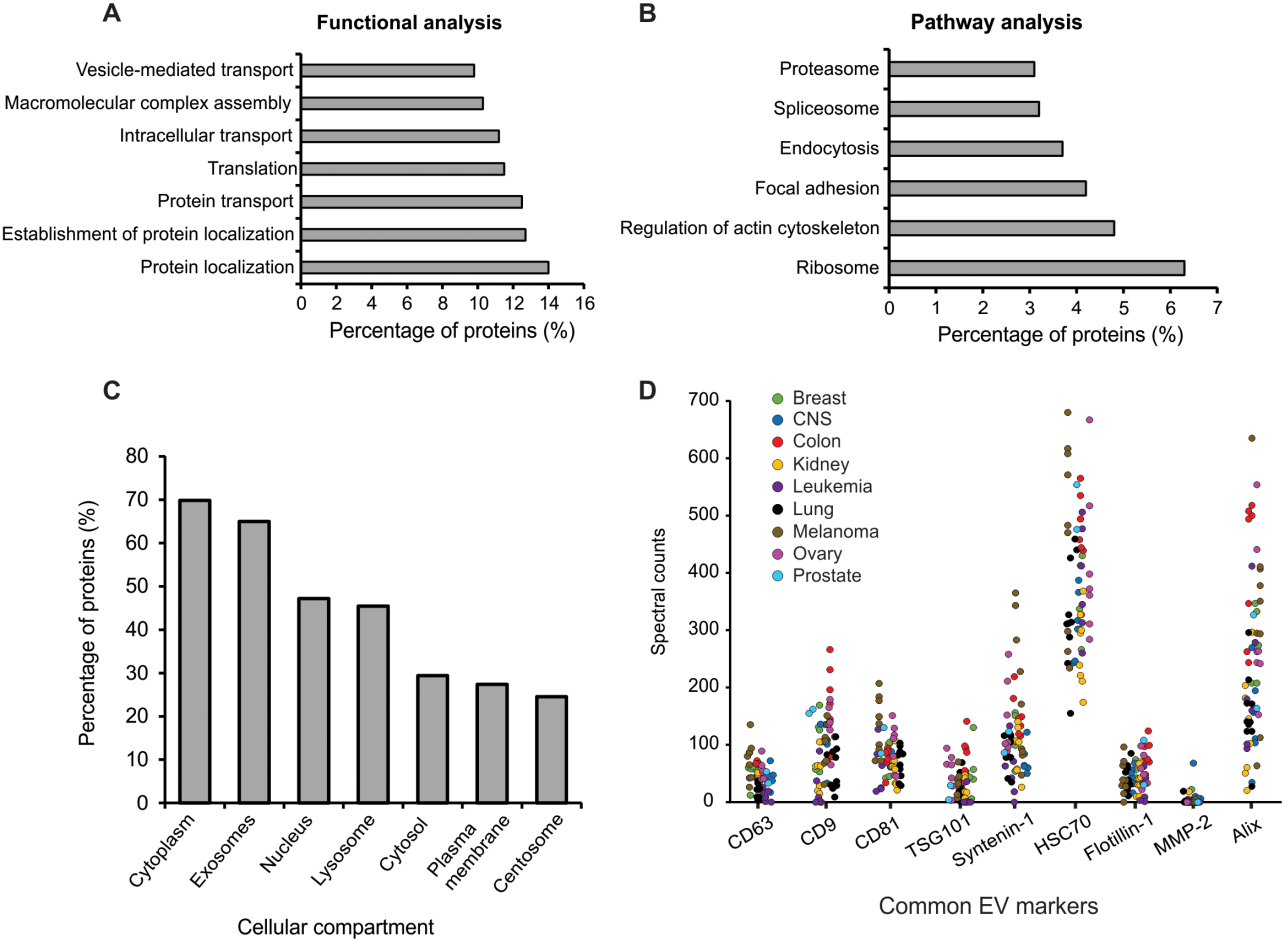
Enrichment analysis of EV proteins identified Proteins identified in the [NCI-60]_stringent_ dataset were used for enrichment analyses. **A.** Functional and **B.** pathway enrichment analysis of EV proteins using the DAVID database (GOTERM_BP_FAT and KEGG_PATHWAY). All terms were significant (p < 0.05) following Benjamini correction. **C.** Subcellular localization enrichment using FunRich. All terms were significant (p < 0.001) following Benjamini correction. **D.** Spectral counts of common vesicle protein markers measured across all NCI-60 EV samples.

To characterize the cargo abundant in the majority of cancer EVs, proteins found in the [NCI-60]_stringent_ dataset were further analyzed. Functional and pathway analyses were conducted using the Database for Annotation, Visualization, and Integrated Discovery (DAVID) v6.7. Not surprisingly, proteins enriched in protein localization, transport, and vesicular functions were identified in our data set (Figure 2A). Many ribosomal proteins involved in translation processes were also enriched, similar to results seen in previous studies [32, 46]. Ribosomal components may facilitate cell-to-cell communication by directly translating mRNAs present in EVs following fusion with target cells. Pathway analysis revealed proteins to be enriched in RNA processing and proteolytic processes, as well as cytoskeletal and endocytic, pathways (Figure 2B). Comparison to the Vesiclepedia database revealed the majority of proteins to have a subcellular localization in endolysosomal or cytoplasmic compartments (Figure 2C). Altogether, these analyses demonstrate an abundance of proteins with recognized functions in protein and vesicle trafficking from the endosomal pathway, and cytoskeletal involvement that likely plays a role in both exosome and microvesicle secretion. The presence of proteins involved in RNA processing suggests an active process of RNA sorting and packaging, and offers insight into how biologically active RNA messages are communicated between cells.

A common method of characterizing EVs relies on the presence of accepted protein markers enriched in vesicle populations. Recent research has described different subpopulations of EVs from cells by identifying vesicle specific protein markers [47]. We compared commonly used exosome and microvesicle markers described across EVs derived from the NCI-60 cells (Figure 2D). Historically, these markers have been considered universal, however, only tetraspanin CD81, Alix, and HSC70 were found across all samples. Tetraspanins CD63 and CD9, as well as TSG101, Syntenin-1, and Flotillin-1 were identified in at least two-thirds of the samples. MMP-2, a previously reported microvesicle marker [32] was found in only 16 EV samples across the panel. Many of these protein markers have historically been used to describe and quantify extracellular vesicles regardless of their cellular localization or method of harvest. However, data presented here show wide variation in the levels of traditional EV markers, with some being completely undetectable in certain EV preparations. We subsequently analyzed EV proteins identified in this study to characterize those common to all cells in the NCI-60 panel. Proteins within this dataset involved in vesicle-mediated transport and protein localization were identified, and were largely enriched in GTPase function, including Rab proteins 1A, 2A, 5C, 6A, 7A, 8A, 10, 11B, and 14 (Supplementary Table S5). These proteins likely represent more universal markers of EVs that future EV researchers should consider for characterization. Notably, as various cells may package these proteins to different degrees within the same number of EVs, the correlation of vesicle quantity across cell lines to any one of these markers is complex. This poses challenges for researchers, as quantitative protein assays such as ELISA or immunoblot analyses are often used to determine vesicle quantity following EV enrichment.

### EV proteomes cluster by tissue type and contain proteins unique to cancer type

**Figure 3:**
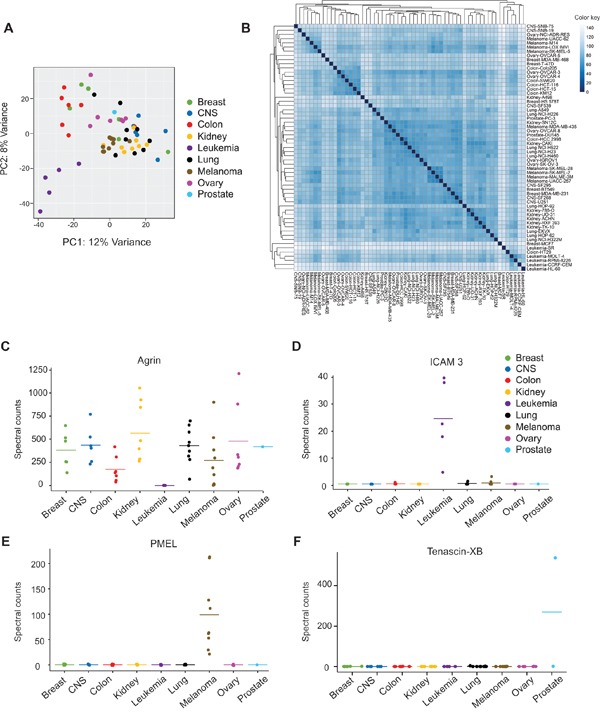
Differential expression of proteins found in NCI-60 cell-derived extracellular vesicles **A.** PCA plot based on variant vesicle proteins across tissue types. **B.** Unsupervised hierarchal clustering of cell lines based on EV proteomic profiles. Spectral count-based differential expression of **C.** Agrin, **D.** Intercellular adhesion molecule (ICAM) 3, **E.** Premelanosome protein (*PMEL*), and **F.** Tenascin-XB. See also Supplementary Figure 1 and Supplementary Table S6.

Next we aimed to compare vesicle proteomes across individual cancer cells in the NCI-60 panel. As extracellular vesicles have been suggested to carry proteins that reflect their progenitor cell, it was hypothesized that EVs released from cells of the same tissue type would share similarities in protein content. Principal component analysis (PCA) demonstrated that EV proteins clustered based on tissue type (Figure 3A). Of note, one leukemia cell line (K562) did not cluster with the remaining cancer EVs (Supplementary Figure 1C). The K562 cell line is an erythroleukemia derived from the pleural effusion of a patient with chronic myelogenous leukemia (CML), and is positive for the Philadelphia translocation on chromosome 22, creating the chimeric BCR/ABL fusion gene. The BCR/ABL gene has been demonstrated to downregulate many cell adhesion molecules [48], which may, in part, explain the divergence from other cell-derived EVs. For instance, in this study, ICAM3, an adhesion molecule abundantly expressed in leukocytes, was found in high levels among all leukemia-derived EVs except the K562 EVs. In light of these findings, the K562 cell line was excluded from all subsequent analyses.

Unsupervised hierarchal clustering was used to further examine the congruity of EV proteomes from cells of the same tissue origin. Samples from colon, kidney, leukemia, lung, and melanoma cancers clustered closely within tissue type (Figure 3B). Breast, CNS, and ovarian cancer EVs demonstrated sub-clustering within each tissue type, suggesting similarities between cancer types that may not be universal across the tissue of origin. Part of these unique clustering patterns may reflect metastatic potential of particular cell lines. For example, highly metastatic breast cancer cells (BT-549 and MDA-MB-231) cluster together away from other non-metastatic breast cell lines.

Strikingly, a number of proteins were observed to be differentially expressed in vesicles secreted from adherent cell lines compared to cells in suspension culture. For example, Agrin (*AGRN*), a basement membrane glycoprotein that contains heparan and chondroitin sulfate residues was absent only in the leukemia-derived EVs (Figure 3C). On the other hand, adhesion molecule ICAM3 was present predominately in EVs secreted from leukemia cancer lines grown in suspension (Figure 3D). As integrin and heparan sulfate proteoglycan receptors have been demonstrated to play a significant role in the uptake of vesicles into cells [16, 49], these observations provide new targets for future studies of tissue-specific mechanisms of vesicular protein trafficking and targeting of EVs to recipient cells.

Recently, a urine-based exosome diagnostic assay has been demonstrated to predict high-grade prostate cancer among men with elevated PSA levels [50]. Likewise, glypican-1, a heparan sulfate proteoglycan, was found to be detected in only cancer-derived exosomes, and was further shown to be correlated with pancreatic cancer progression in patients, providing a non-invasive early diagnostic tool for pancreatic cancer [23]. In the NCI-60 panel, glypican-1 was identified in only 35 of 60 cancer EV isolates. This suggests an even stronger likelihood of novel protein markers identified across *all* samples in this study (or all samples within a tissue-type) to be useful as early diagnostic markers of cancers. Furthermore, the evidence showing differences in EV contents from circulating cells (leukocytes) compared to basement membrane-adhered cells is particularly valuable when considering liquid biopsy techniques to isolate vesicles, as proteins such as ICAM3 could serve as a tool to distinguish blood cell-borne EVs from those secreted into circulation by organ-derived or metastatic cells.

Extracellular vesicle-based liquid biopsies also carry the potential for early detection of cancer cells that normally have limited access to blood circulation. The proteomic analysis of NCI-60 EVs confirmed the presence of premelanosome protein (*PMEL*) in vesicles secreted from all melanoma cancer cell types (Figure 3E). This melanocyte-specific transmembrane glycoprotein has previously been shown to be sorted into endosomes for exosomal secretion [51]. As melanocytes are ordinarily confined to the epidermal layers of the skin, access to deeper blood vessels is not usually achieved unless vertical growth of cancerous cells occurs. Therefore, a circulating (plasma) melanocyte-specific exosomal protein marker could serve as an early indication of various types of invasive melanoma growth.

Moreover, several vesicular proteins were identified in a very small population of cancer lines. For instance, Tenascin XB (*TNXB*), an extracellular matrix glycoprotein was found in abundant levels only in PC-3 cells, a high grade prostate adenocarcinoma line. Although Tenascin XB was not identified in whole cell proteomic profiling from PC-3 cells [28], relatively high transcript levels of this protein in PC-3 cells have been described previously [52]. Another EV protein, periostin was recently identified as a metastatic breast cancer vesicular marker [22]. In our study, the presence of periostin was confirmed in both metastatic breast cancer lines (MDA-MB-231 and HS 578T), but was not found in other non-metastatic breast cancer-derived EVs. Additional proteins including raftilin (a lipid-raft regulating protein), fibulin-7 (an adhesion molecule), and plasminogen activator inhibitor 1 (a serine protease inhibitor that has previously been implicated in aggressive tumor growth [53, 54]) were exclusively found in metastatic breast cancer EVs. Likewise, latent-TGFβ-binding protein-1 was identified preferentially in lung cancer cells with high invasive capacity (A549, HOP-62, and HOP-92) [55]. In all, over 1,500 proteins were found to be differentially expressed across the 60 EV samples (Supplementary Table S6). Interestingly, comparison of whole cell protein expression reported by Gholami et al. to EV expression in Figure 3C-3E [28] revealed these differentially expressed proteins to be largely conserved between cell and vesicle isolates. For instance, ICAM3 was chiefly absent in non-leukemic tumor cells, while Agrin was underrepresented in leukemia-derived cells (Supplementary Figure S2). Likewise, PMEL was found in substantial levels in melanoma cell lines compared to other cancer cells. These findings suggest that differentially expressed proteins found in cancer EVs may reflect cellular phenotypes. Furthermore, the specificity of protein content in vesicles from individual cancer cell types promises great potential in further investigation of these novel markers for early cancer detection and prognostic monitoring.

### Conserved vesicular proteins are correlated with EV secretion

Recently, we described relative extracellular vesicle secretion quantities across the NCI-60 panel [41]. As many EV proteins in samples across the study were identified in different quantities, we hypothesized that some of these proteins are likely involved in vesicle biogenesis and therefore correlate to the total number of vesicles secreted by cells. To investigate proteins involved in common pathways of EV formation, levels of proteins in the [NCI-60]_stringent_ were compared to previously collected vesicle secretion quantities [41] (particles per cell; Supplementary Table S7) by weighted gene coexpression network analysis (WGCNA).

**Figure 4:**
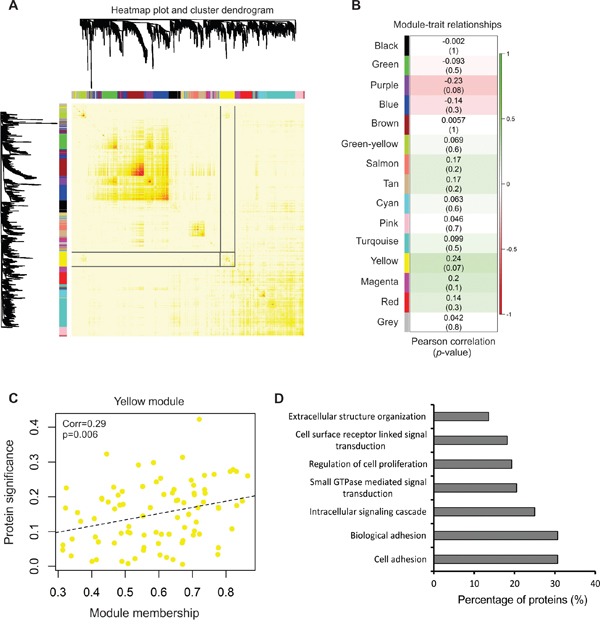
Network analysis of protein content with vesicle secretion **A.** Network heatmap plot of topological overlap depicting protein dendrogram and module assignment. Targeted yellow module is highlighted. See also Supplementary Figure 3. **B.** Heatmap of module-trait correlation containing correlation and relevant *p*-values of modules detected. See also Supplementary Table S7 and S8. **C.** Scatter plot of membership in the yellow module and protein significance corresponding to vesicle secretion quantities across the NCI-60. A significant positive correlation between protein significance and module membership was determined (*p* = 0.006). **D.** GOTERM_BP_FAT analysis of biological processes enriched in the yellow module protein dataset.

In this analysis, hierarchal clustering of proteins (Figure 4A) demonstrated inter-related expression patterns and produced 15 clusters of highly related proteins (modules) that were detected by dynamic tree cut, an optimal method used to detect clusters of data within a dendrogram [56]. Modules were then correlated to vesicle secretion patterns across the panel (Supplementary Table S8). The yellow module containing 88 proteins was most significantly correlated with particle secretion, and therefore served as our target protein cluster (Figure 4B).

Here, protein significance is defined as the correlation of the protein expression profile across the NCI-60 panel with particle secretion levels. Module membership further measures the correlation of protein expression patterns across the members of the yellow module. We found protein significance and module membership to be positively correlated in the yellow module (*p* = 0.006) (Figure 4C). These findings suggest that proteins clustered into the module show interconnected profiles of expression in vesicles that positively correlated with the number of vesicles secreted by cells.

Enrichment analysis of the yellow module demonstrated proteins were significantly enriched in cell adhesion and growth, GTPase activity, and cell surface receptor signaling (Figure 4D), and included CD63, CD81, VAMP3, syntenin-1, and SEC22B, among other vesicular proteins. Strikingly, 25 of the yellow module proteins were identified in EVs from every cancer cell in the panel (Table 1), supporting the hypothesis that commonly identified EV components likely play a role in EV biogenesis. In light of the variation in current EV markers seen (Figure 2D), these represent important proteins that could more accurately compare vesicle quantities across a diversity of cell lines and certainly warrant future investigation.

**Table 1:** Common cancer proteins associated with vesicle secretion

Protein ID	Gene symbol	Protein name	Average spectral count per sample
**P07355**	*ANXA2*	Annexin A2	288.53
**P18085**	*ARF4*	ADP-ribosylation factor 4	18.05
**P35613**	*BSG*	Basigin	79.12
**P13987**	*CD59*	CD59 glycoprotein	34.40
**P60033**	*CD81*	CD81 antigen	83.18
**P05534**	*HLA-A*	HLA class I histocompatibility antigen, A-24 alpha chain	92.82
**P01112**	*HRAS*	GTPase HRas	40.58
**P0DMV9**	*HSPA1B*	Heat shock 70 kDa protein 1B	142.08
**P04792**	*HSPB1*	Heat shock protein beta-1	74.40
**P05556**	*ITGB1*	Integrin beta-1	168.10
**Q08380**	*LGALS3BP*	Galectin-3-binding protein	550.05
**P08590**	*MYL3*	Myosin light chain 3	10.32
**P60660**	*MYL6*	Myosin light polypeptide 6	72.50
**P22392**	*NME2*	Nucleoside diphosphate kinase B	75.72
**P01111**	*NRAS*	GTPase NRas	7.08
**P51148**	*RAB5C*	Ras-related protein Rab-5C	51.18
**P51149**	*RAB7A*	Ras-related protein Rab-7a	64.45
**P63000**	*RAC1*	Ras-related C3 botulinum toxin substrate 1	43.45
**P11234**	*RALB*	Ras-related protein Ral-B	38.67
**P61224**	*RAP1B*	Ras-related protein Rap-1b	111.35
**P61586**	*RHOA*	Transforming protein RhoA	81.78
**O75396**	*SEC22B*	Vesicle-trafficking protein SEC22b	21.85
**P37802**	*TAGLN2*	Transgelin-2	87.45
**P61077**	*UBE2D3*	Ubiquitin-conjugating enzyme E2 D3	7.58
**Q15836**	*VAMP3*	Vesicle-associated membrane protein 3	28.08

### Cancer vesicle proteomes reflect the molecular composition of progenitor cells

**Figure 5:**
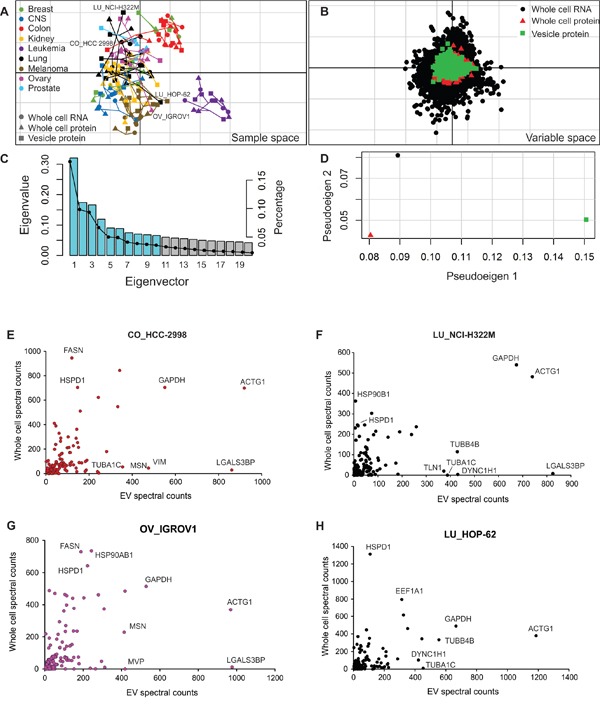
Comparison of vesicular proteome with cellular proteome and transcriptome **A.** Co-inertia analysis of cellular proteome and transcriptome with vesicular proteome across the NCI-60 panel. Vectors represent the proportional divergence between data sets within each cell line. **B.** Coordinates of the proteins or RNA from each data set plotted in the same orientation as the co-inertia analysis. **C.** Histogram of eigenvalues. Blue bars represent the absolute values of eigenvalues retained in the analysis. Black dots represent the proportion of variance found within each eigenvector. **D.** Plot of pseudo-eigenvalues space, indicating the variance of eigenvalues contributed by each of the three datasets. Comparison of expression levels of core vesicular proteins with whole cell protein expression from representative cells lines: **E.** HCC-2993 (colon); **F.** NCI-H322M (lung); **G.** IGROV1 (ovary); and H. HOP-62 (lung). See also Supplementary Table S9.

Given the clinical utility of using extracellular vesicles for cancer diagnostics, we investigated the relationships between EV protein composition and whole cell content. Previously, cellular protein and transcript expression profiles were compared using co-inertia analysis (CIA) to examine the concordance between these molecular datasets across the NCI-60 panel [28]. Here vesicle protein levels were similarly compared to cellular protein and RNA expression. In Figure 5A, each of the three datasets (vesicle proteome, cellular proteome, and cellular transcriptome) is plotted for individual cell lines. Markers represent the relative position of a cell line in respective proteome or transcriptome space, where the divergence of datasets is delineated by connecting vectors. Molecular factors driving tissue-dependent clustering were plotted as overlapping protein (cell and vesicle) and RNA transcript data in the same orientation (Figure 5B). In variable space, RNA or protein coordinates farther from the origin are more highly expressed in cell lines projected in the same direction. Whole cell RNA profiling showed the strongest ability to differentiate samples, demonstrated by farther distances of RNAs from the origin in variable space. However, vesicle protein was observed to show similar magnitudes of projection from the origin as whole cell protein, demonstrating the ability of EV proteins to reflect cell protein profiling. A histogram of eigenvalues (Figure 5C) demonstrated that the first and second co-inertia axes represented 49% of the total variance (sum of the eigenvalues) seen in the datasets plotted, accounting for 31% and 18% of the variance respectively. The three datasets were also examined to consider how much variance of the eigenvalues was contributed by each dataset (Figure 5D). No single dataset contributed to both co-inertia axes alone, indicating that the analysis examining the relationships between vesicle proteomes and cellular transcriptomic and proteomic profiles was dependent on all datasets.

In general, melanoma, leukemia and colon cells demonstrated tissue-type clustering based on their unique proteomic and transcriptomic profiles, similar to clusters previously observed (Figure 3A-3B). The vesicle protein dataset showed considerable association with the whole cell molecular profiles, indicated by the short and randomly oriented arrows (Figure 5A). Overall, the similarity of vesicle protein to cellular RNA and protein resulted in RV coefficients of 0.63 and 0.57, respectively. Notably, several cell lines demonstrated greater variation between vesicle protein and whole cell profiles, including two lung lines (NCI-H322M and HOP-62), one colon cell (HCC-2998), and an ovarian cell (IGROV1). To more closely examine proteins found in discordance between whole cell and EV proteomes, proteins found in the core vesicle proteome were compared to reported cellular expression data [28]. In total, 144 proteins identified in the core vesicle proteome in this study were similarly identified in cell lines, as reported by Gholami et al. Comparison of expression levels between whole cell and vesicular isolates revealed several outlying proteins enriched in vesicles or cells alone (Figure 5E–5H and Supplementary Table S9). While proteins such as Actin (ACTG1) or Glyceraldehyde-3-phosphate dehydrogenase (GAPDH) were found abundantly in both cells and EVs, tubulin proteins (TUBB4B and TUBA1C) were enriched in lung cancer vesicles compared to cells, and actin-binding protein Moesin (MSN) was enriched in vesicles from IGROV1 and HCC-2998 cells. Strikingly, Galectin-3-binding protein (LGALS3BP) was enriched in multiple EV isolates compared to respective progenitor cells. These proteins probably represent targets of selective vesicular packaging, and future focus will likely advance our understanding of vesicular trafficking mechanisms.

Altogether, the congruency of tissue dependent clusters across datasets and resemblance between the vesicle and cellular molecular profiles substantiates the ability of EV profiles to discriminate between tissue types and supports a significant clinical value of EVs as circulating biomarkers. As EVs can closely reflect the molecular profile of their cellular origin, they therefore represent circulating biological archetypes of healthy or diseased cells.

## CONCLUSIONS

In the past decade, it has become clear that extracellular vesicles are an important component of cellular communication informing fields including cancer biology, immunology, and virology. The data here provide a means to better understand the protein cargo involved in this ubiquitous messaging system with implications for further use in clinical diagnostic practice. In the characterization of vesicular protein contents from the NCI-60 cells, we have expanded the list of known vesicle proteins by nearly twenty percent (based on the Vesiclepedia database of characterized vesicle proteins), significantly growing our foundation of knowledge of the nature of this communication system. Of these proteins identified, 213 were common to all cell lines. This core EV proteome likely represents conserved structural and signaling components of vesicles, and molecular factors involved in vesicle biogenesis and secretion. In support of this, 25 of the 213 proteins were found to be positively correlated with EV secretion levels. One of the largest obstacles currently faced by EV researchers is accurately defining these vesicles subtypes. The data presented in this study offer a substantial step forward in the ability to define EV populations, and to enrich vesicles for studies in all areas of EV research. As our ability to isolate distinct vesicle sub-populations continues to improve, this substantial database will likely provide an important backbone for understanding more unique vesicle contents.

In this study, critical differences in extracellular vesicle contents were also identified which may directly lead to the discovery of biomarkers of cancer, ultimately affecting diagnosis and prognosis of the increasingly prevalent disease. As emerging large-scale “omic” datasets progressively demonstrate significant potential in the future of individualized medicine, it is expected that extracellular vesicles will play an important role in modern health and disease-state surveillance. In this study, the close resemblance of vesicle and cellular molecular profiles suggests that extracellular vesicles reliably represent their progenitor cells. They are therefore excellent candidates for bearing biomarkers, and will increasingly find a place amongst clinical strategies for combating diseases like cancer.

## EXPERIMENTAL METHODS

### Cell culture

Sixty cell lines from the National Cancer Institute (NCI-60) were acquired from the NCI Developmental Therapeutics Program. Cells were seeded based on growth rate (doubling time) to achieve 80-90% confluence after two to three days. Cells were cultured in RPMI-1640 medium (Lonza, 12-702Q) supplemented with 10% fetal bovine serum (FBS; Seradigm, 1400-500), 2 mM L-glutamine (Corning, 25-005-CI), 100 units penicillin/streptomycin (Corning, 30-002-CI), and 100 units:100 μg/mL:0.25 μg/mL antibiotic/antimycotic (Corning, 30-002-CI). Following the two to three day culture, complete medium was aspirated and cells were washed with warmed sterile phosphate buffered saline (PBS). To minimize contaminating serum proteins in downstream mass spectrometry analysis and aid in the identification of lower abundance EV proteins, cells were cultured in serum-free medium for a further 48 hours before EV enrichment. Previous results have demonstrated that EVs harvested using this method are as pure as sucrose-cushion purified vesicles [43].

### EV enrichment and protein quantification

Following the 48 hour serum-free culture, medium was harvested. Cell viability at the time of harvest was measured by counting cells stained with 0.2% trypan blue in PBS (Sigma, T8154) or AO/PI (Nexcelom Bioscience, CS2-0106) with an automated cell counter (Cellometer Vision, v2.1.4.2, Nexcelom Bioscience). We have previously demonstrated that cell viability does not significantly contribute to the number or size of particles secreted by the NCI-60 cells when viabilities are maintained greater than 85% [41]. In this study, cell viabilities following the forty-eight hour serum-free culture approximated those observed previously in complete medium. EVs were enriched using the ExtraPEG method previously described [43]. Briefly, cell-conditioned medium was centrifuged at 500 g for five minutes to pellet and discard cells, followed by 2,000 g for 30 minutes to remove cellular debris. Supernatant was pooled from several flasks to amass sufficient material (200-500 mL) for downstream proteomic analysis. A 1:1 volume of 2X PEG solution [16% (w/v) polyethylene glycol, 1 M NaCl] was added. Samples were inverted to mix, then incubated overnight. The next day, the medium/PEG mixture was centrifuged at 3,214 g for one hour. Crude vesicle pellets were resuspended in 1 mL of particle-free PBS and re-pelleted by ultracentrifugation at 100,000 g for 70 minutes. Final pellets were lysed in strong lysis buffer [5% SDS, 10 mM EDTA, 120 mM Tris-HCl pH 6.8, 2.5% β-mercaptoethanol, 8 M urea] with the addition of HALT protease inhibitor (Thermo, 78438). Vesicular protein was quantified using the fluorescence-based EZQ™ Kit (Thermo, R33200).

### SDS PAGE and in-gel digestion

For protein purification and separation by sodium dodecyl sulfate polyacrylamide gel electrophoresis (SDS-PAGE), 20 μg of vesicular protein from each sample was loaded into a 4-20% polyacrylamide gel (Lonza, 59511). Following electrophoresis, gels were fixed and Coomassie-stained as previously detailed [57]. Samples were fractionated by cutting gel lanes into five sections. Sections were subsequently subdivided into 1 mm^3^ cubes before trypsin-digesting as described [43].

### Protein identification and quantification

Following protein digestion, samples were submitted to the Florida State University Translational Science Laboratory for liquid chromatography tandem mass spectrometry (LC-MS/MS) analysis. The digest was freeze-dried and resuspended in 30 μL 0.1% FA and analyzed by liquid chromatography tandem mass spectrometry (LC-MS). An externally calibrated Thermo LTQ Orbitrap Velos nLC-ESI-LTQ-Orbitrap (high-resolution electrospray tandem mass spectrometer) was used with the following parameters: A 2 cm trap column of 100 μm internal diameter (i.d.) (SC001 Easy Column from Thermo-scientific) was followed by a 10 cm analytical column of 75 μm i.d. (SC200 Easy Column from Thermo-scientific). Both trap column and analytical column had C18-AQ packaging. Separation was carried out using Easy nanoLC II (Thermo-Scientific) with a continuous, vented column configuration. A 5 μL sample was aspirated into a 20 μL sample loop and loaded onto the trap. The flow rate was set to 300 nL/min for separation on the analytical column. Mobile phase A was composed of 99.9 H_2_O (EMD Omni Solvent), and 0.1% formic acid, and mobile phase B was composed of 99.9% ACN and 0.1% formic acid. A 1 hour linear gradient from 0% to 45% B was performed. The LC eluent was directly nano-sprayed into an LTQ Orbitrap Velos mass spectrometer (Thermo Scientific). During the chromatographic separation, the LTQ Orbitrap Velos was operated in a data-dependent mode and under direct control of the Xcalibur software (Thermo Scientific). The mass spectrometry data were acquired using the following parameters: 10 data-dependent collisional-induced-dissociation (CID) MS/MS scans per full scan. All measurements were performed at room temperature and three technical replicates were run for each sample.

Raw data collected from each of five fractions were pooled by sample and analyzed using MaxQuant (v1.5.3.30). The mass spectrometry data were analyzed using the integrated Andromeda peptide search engine and a recent (March 2016) UniProt knowledgebase reviewed (Swiss-prot) human protein database. The database was appended with a list of common contaminants in MaxQuant, and search parameters used were either the default settings for this version of the software, or as follows. Instrument type was set to Orbitrap, using label free quantitation, and first search peptide tolerance set to 10 ppm, and main search peptide tolerance at 4.5 ppm. Digestion mode was set specific for trypsin, with a maximum of two missed cleavages. Fixed modifications included only carbamidomethyl (C), and variable modifications included oxidation (M), N-terminal acetylation, and phosphorylation (STY). A maximum of five modifications were allowed per peptide. False discovery rate was set to 0.01. The mass spectrometry proteomics data have been deposited to the ProteomeXchange Consortium [58] via the PRIDE partner repository.

Primary analyses were conducted with the MaxQuant output data set (NCI-60). This dataset represents all proteins identified in the study. To examine proteins found in the majority of samples that reflect a more commonly shared cancer EV proteome, a more stringent protein set was used, and defined as [NCI-60]_stringent_. This data set includes only proteins found in at least two thirds of all EV samples.

### Differential expression as a function of tissue origin

For differential expression and network analyses, raw spectral count data were exported from the MaxQuant software and imported into R statistical framework. The spectral count based method has been demonstrated to provide a reliable representation of protein abundance with a linear dynamic range over several orders of magnitude with similar sensitivity as ion peak intensity quantitation [59–61]. Utilization of spectral counts was necessary for our downstream pipeline analyses using DeSeq2 for the identification of differentially expressed proteins and normalization for WGCNA and co-inertia analyses. Raw peak intensity data are reported in Supplementary Table 1.

DESeq2 is a package originally designed for analyzing read counts from RNA-sequencing datasets and recently used to analyze other forms of biological count data, including LC-MS/MS spectral count data [62]. Normalization of spectral counts with DESeq2 considerably reduced the difference in sample depth between samples (Supplementary Figure 1A and 1B). PCA analysis of the top 500 variant proteins revealed one sample with substantially different protein expression profiles (K562) (Supplementary Figure 1C), which was removed from further analysis. After normalization, a likelihood ratio test was performed across all samples in DESeq2 to identify proteins with significantly different patterns of expression between samples of different tissue origin. Proteins that returned an FDR-adjusted *p*-value (q value) of less than 0.05 were considered significant.

### Weighted gene coexpression network analysis (WGCNA)

The WCGNA was originally implemented for the analysis of transcriptomic microarray data, but has recently been used to analyze RNA-seq and LC-MS/MS proteomics datasets [63, 64]. To identify proteins that are associated with the regulation of vesicle secretion, a network analysis was performed with the WGCNA package in the R statistical framework [65]. Tutorials for WGCNA can be found at http://labs.genetics.ucla.edu/horvath/CoexpressionNetwork/. WGCNA is a multi-step analysis which involves: 1) generating a coexpression matrix for gene (or protein) expression, 2) transforming protein correlations into a adjacency matrix for network construction, 3) grouping together proteins (modules) that show high co-correlation, and 4) correlating an eigenprotein, the first principle component of each module, with a biological trait of interest. Importantly, WGCNA avoids the extensive FDR-correction needed to control for false positives in protein-wise differential expression analyses, as there are many fewer modules in the entire network than proteins.

Normalized [NCI-60]_stringent_ count data were transformed in DESeq2 with a variance stabilizing transformation using the “VST” function, and used as input for WGCNA. The resulting count matrix was used as input for WGCNA. We used the one-step network construction and module detection function “blockwiseModules.” Modules of co-regulated proteins across the vesicle protein abundance profiles of the NCI-60 cell lines were identified by first grouping proteins (nodes) together into clusters of highly co-correlated members based on their degree of co-correlation (edges). For measuring the coexpression similarity between proteins, we used a signed Pearson correlation. For adjacency matrix construction, soft thresholding was used. The scale-free fitting index approached a local maximum at β = 12 (Supplementary Figure S3). This threshold approximately achieves scale free topology, meaning that the node degree distribution approximately follows the power law. Therefore, the co-correlation matrix was raised to the power of 12 to create the adjacency matrix. Finally, the adjacency matrix was converted into a topological overlap matrix, and the corresponding dissimilarities (1 – the topological overlap matrix) was used for module detection via hierarchical clustering. The minimum number of proteins required per module was set to 30, and the mergeCutHeight was set to 0.25, such that sufficiently similar modules were merged revealing 15 modules. Module eigenproteins, a synthetic measure of module belonging, were then related to the quantity of vesicles secreted per cell previously collected [28] by calculating Pearson correlation coefficients. The module most significantly associated with particle secretion was retained for enrichment analysis.

### Comparison between vesicle and whole-cell molecular profiles

Co-inertia analysis (CIA; [66, 67]) was used to examine similarities between the vesicle proteome and cellular proteome and transcriptome across the NCI-60 cell lines. Microarray gene expression data was downloaded from the NCBI Gene Expression Omnibus [68] (accession number GSE32474; [69, 70]). Whole-cell proteome data was obtained from the NCI60 proteome resource (http://proteomics.wzw.tum.de/nci60; [28]). Spectral count data from the whole-cell dataset was processed analogously to the EV proteome dataset for comparability: raw spectral count data published by Moghaddas Gholami et al. were normalized with DESeq2 following removal of all proteins not found with at least one count in 40 or more samples, and further transformed with the VST function. NCI-60 cell lines not included in all three datasets were excluded from this analysis, leaving 56 samples in total. Differences between samples and molecular assays were analyzed and visualized with co-inertia analysis using the R omicade4 package [71] in the R statistical framework.

### Protein set enrichment analysis

To identify proteins previously found in EVs, the Vesiclepedia database of vesicular proteins was downloaded (Version 3, 9 Jan 2015) from microvesicles.org/download. The Database for Annotation, Visualization, and Integrated Discovery (DAVID) v6.7 [72, 73] was used for functional (GOTERM_BP_FAT) and pathway (KEGG_PATHWAY) analyses of proteins found in the [NCI-60]_stringent_ dataset (Figure 2A-2B). Enrichment of cellular compartment terms (Figure 2C) was analyzed using FunRich v3 [74].

For further interpretation of the target WGCNA module, enrichment of biological processes was performed using DAVID: GOTERM_BP_FAT. Proteins entered into the WGCNA analysis were used as the background dataset, and proteins within the target module were used as the target list. All terms with a *p*-value (Benjamini or Benjamini-Hochberg adjusted) less than 0.05 were considered significant and ranked by the number of proteins identified in the group.

## SUPPLEMENTARY FIGURES AND TABLES





















## References

[1] Bobrie A, Colombo M, Raposo G, Théry C (2011). Exosome secretion: molecular mechanisms and roles in immune responses. Traffic.

[2] Théry C, Zitvogel L, Amigorena S (2002). Exosomes: composition, biogenesis and function. Nat Rev Immunol.

[3] Mathivanan S, Ji H, Simpson RJ (2010). Exosomes: extracellular organelles important in intercellular communication. J Proteomics.

[4] Théry C (2011). Exosomes: secreted vesicles and intercellular communications. F1000 Biol Rep.

[5] Raposo G, Stoorvogel W (2013). Extracellular vesicles: exosomes, microvesicles, and friends. J Cell Biol.

[6] Zhang X, Yuan X, Shi H, Wu L, Qian H, Xu W (2015). Exosomes in cancer: small particle, big player. J Hematol Oncol.

[7] Park JE, Tan HS, Datta A, Lai RC, Zhang H, Meng W, Lim SK, Sze SK (2010). Hypoxic tumor cell modulates its microenvironment to enhance angiogenic and metastatic potential by secretion of proteins and exosomes. Mol Cell Proteomics.

[8] Meckes DG, Shair KH, Marquitz AR, Kung CP, Edwards RH, Raab-Traub N (2010). Human tumor virus utilizes exosomes for intercellular communication. Proc Natl Acad Sci U S A.

[9] Meckes DG, Raab-Traub N (2011). Microvesicles and viral infection. J Virol.

[10] Meckes DG (2015). Exosomal communication goes viral. J Virol.

[11] Pegtel DM, Cosmopoulos K, Thorley-Lawson DA, van Eijndhoven MA, Hopmans ES, Lindenberg JL, de Gruijl TD, Würdinger T, Middeldorp JM (2010). Functional delivery of viral miRNAs via exosomes. Proc Natl Acad Sci U S A.

[12] Costa-Silva B, Aiello NM, Ocean AJ, Singh S, Zhang H, Thakur BK, Becker A, Hoshino A, Mark MT, Molina H, Xiang J, Zhang T, Theilen TM (2015). Pancreatic cancer exosomes initiate pre-metastatic niche formation in the liver. Nat Cell Biol.

[13] Kim HK, Song KS, Park YS, Kang YH, Lee YJ, Lee KR, Ryu KW, Bae JM, Kim S (2003). Elevated levels of circulating platelet microparticles, VEGF, IL-6 and RANTES in patients with gastric cancer: possible role of a metastasis predictor. Eur J Cancer.

[14] Baran J, Baj-Krzyworzeka M, Weglarczyk K, Szatanek R, Zembala M, Barbasz J, Czupryna A, Szczepanik A (2010). Circulating tumour-derived microvesicles in plasma of gastric cancer patients. Cancer Immunol Immunother.

[15] Silva J, Garcia V, Rodriguez M, Compte M, Cisneros E, Veguillas P, Garcia JM, Dominguez G, Campos-Martin Y, Cuevas J, Peña C, Herrera M, Diaz R (2012). Analysis of exosome release and its prognostic value in human colorectal cancer. Genes Chromosomes Cancer.

[16] Hoshino A, Costa-Silva B, Shen TL, Rodrigues G, Hashimoto A, Tesic Mark M, Molina H, Kohsaka S, Di Giannatale A, Ceder S, Singh S, Williams C, Soplop N (2015). Tumour exosome integrins determine organotropic metastasis. Nature.

[17] Kalluri R (2016). The biology and function of exosomes in cancer. J Clin Invest.

[18] Wilbur J (2008). Prostate cancer screening: the continuing controversy. Am Fam Physician.

[19] Fritsche HA, Bast RC (1998). CA 125 in ovarian cancer: advances and controversy. Clin Chem.

[20] Yang GB (2015). Clinical value of serum cancer antigen 19-9 as a tumor screening marker among healthy individuals. J BUON.

[21] Meckes DG, Gunawardena HP, Dekroon RM, Heaton PR, Edwards RH, Ozgur S, Griffith JD, Damania B, Raab-Traub N (2013). Modulation of B-cell exosome proteins by gamma herpesvirus infection. Proc Natl Acad Sci U S A.

[22] Vardaki I, Ceder S, Rutishauser D, Baltatzis G, Foukakis T, Panaretakis T (2016). Periostin is identified as a putative metastatic marker in breast cancer-derived exosomes. Oncotarget.

[23] Melo SA, Luecke LB, Kahlert C, Fernandez AF, Gammon ST, Kaye J, LeBleu VS, Mittendorf EA, Weitz J, Rahbari N, Reissfelder C, Pilarsky C, Fraga MF (2015). Glypican-1 identifies cancer exosomes and detects early pancreatic cancer. Nature.

[24] Boukouris S, Mathivanan S (2015). Exosomes in bodily fluids are a highly stable resource of disease biomarkers. Proteomics Clin Appl.

[25] Shoemaker RH (2006). The NCI60 human tumour cell line anticancer drug screen. Nat Rev Cancer.

[26] Weinstein JN (2006). Spotlight on molecular profiling: “Integromic” analysis of the NCI-60 cancer cell lines. Mol Cancer Ther.

[27] Weinstein JN (2004). Integromic analysis of the NCI-60 cancer cell lines. Breast Dis.

[28] Moghaddas Gholami A, Hahne H, Wu Z, Auer FJ, Meng C, Wilhelm M, Kuster B (2013). Global proteome analysis of the NCI-60 cell line panel. Cell Rep.

[29] Sinha A, Ignatchenko V, Ignatchenko A, Mejia-Guerrero S, Kislinger T (2014). In-depth proteomic analyses of ovarian cancer cell line exosomes reveals differential enrichment of functional categories compared to the NCI 60 proteome. Biochem Biophys Res Commun.

[30] Staubach S, Razawi H, Hanisch FG (2009). Proteomics of MUC1-containing lipid rafts from plasma membranes and exosomes of human breast carcinoma cells MCF-7. Proteomics.

[31] Ji H, Greening DW, Barnes TW, Lim JW, Tauro BJ, Rai A, Xu R, Adda C, Mathivanan S, Zhao W, Xue Y, Xu T, Zhu HJ (2013). Proteome profiling of exosomes derived from human primary and metastatic colorectal cancer cells reveal differential expression of key metastatic factors and signal transduction components. Proteomics.

[32] Keerthikumar S, Gangoda L, Liem M, Fonseka P, Atukorala I, Ozcitti C, Mechler A, Adda CG, Ang CS, Mathivanan S (2015). Proteogenomic analysis reveals exosomes are more oncogenic than ectosomes. Oncotarget.

[33] Valenzuela MM, Ferguson Bennit HR, Gonda A, Diaz Osterman CJ, Hibma A, Khan S, Wall NR (2015). Exosomes Secreted from Human Cancer Cell Lines Contain Inhibitors of Apoptosis (IAP). Cancer Microenviron.

[34] Kong JN, He Q, Wang G, Dasgupta S, Dinkins MB, Zhu G, Kim A, Spassieva S, Bieberich E (2015). Guggulsterone and bexarotene induce secretion of exosome-associated breast cancer resistance protein and reduce doxorubicin resistance in MDA-MB-231 cells. Int J Cancer.

[35] Shedden K, Xie XT, Chandaroy P, Chang YT, Rosania GR (2003). Expulsion of small molecules in vesicles shed by cancer cells: association with gene expression and chemosensitivity profiles. Cancer Res.

[36] Clayton A, Mitchell JP, Court J, Linnane S, Mason MD, Tabi Z (2008). Human tumor-derived exosomes down-modulate NKG2D expression. J Immunol.

[37] Sung BH, Ketova T, Hoshino D, Zijlstra A, Weaver AM (2015). Directional cell movement through tissues is controlled by exosome secretion. Nat Commun.

[38] Webber JP, Spary LK, Sanders AJ, Chowdhury R, Jiang WG, Steadman R, Wymant J, Jones AT, Kynaston H, Mason MD, Tabi Z, Clayton A (2015). Differentiation of tumour-promoting stromal myofibroblasts by cancer exosomes. Oncogene.

[39] Phuyal S, Hessvik NP, Skotland T, Sandvig K, Llorente A (2014). Regulation of exosome release by glycosphingolipids and flotillins. FEBS J.

[40] Kosaka N, Iguchi H, Hagiwara K, Yoshioka Y, Takeshita F, Ochiya T (2013). Neutral sphingomyelinase 2 (nSMase2)-dependent exosomal transfer of angiogenic microRNAs regulate cancer cell metastasis. J Biol Chem.

[41] Hurwitz SN, Conlon MM, Rider MA, Brownstein NC, Meckes DG (2016). Nanoparticle analysis sheds budding insights into genetic drivers of extracellular vesicle biogenesis. J Extracell Vesicles.

[42] Webber J, Clayton A (2013). How pure are your vesiclesŒ. J Extracell Vesicles.

[43] Rider MA, Hurwitz SN, Meckes DG (2016). ExtraPEG: A Polyethylene Glycol-Based Method for Enrichment of Extracellular Vesicles. Scientific Reports.

[44] Kalra H, Simpson RJ, Ji H, Aikawa E, Altevogt P, Askenase P, Bond VC, Borràs FE, Breakefield X, Budnik V, Buzas E, Camussi G, Clayton A (2012). Vesiclepedia: a compendium for extracellular vesicles with continuous community annotation. PLoS Biol.

[45] Liu Y, Cao X (2016). Organotropic metastasis: role of tumor exosomes. Cell Res.

[46] Zhu Y, Chen X, Pan Q, Wang Y, Su S, Jiang C, Li Y, Xu N, Wu L, Lou X, Liu S (2015). A Comprehensive Proteomics Analysis Reveals a Secretory Path- and Status-Dependent Signature of Exosomes Released from Tumor-Associated Macrophages. J Proteome Res.

[47] Kowal J, Arras G, Colombo M, Jouve M, Morath JP, Primdal-Bengtson B, Dingli F, Loew D, Tkach M, Théry C (2016). Proteomic comparison defines novel markers to characterize heterogeneous populations of extracellular vesicle subtypes. Proc Natl Acad Sci U S A.

[48] Jongen-Lavrencic M, Salesse S, Delwel R, Verfaillie CM (2005). BCR/ABL-mediated downregulation of genes implicated in cell adhesion and motility leads to impaired migration toward CCR7 ligands CCL19 and CCL21 in primary BCR/ABL-positive cells. Leukemia.

[49] Christianson HC, Svensson KJ, van Kuppevelt TH, Li JP, Belting M (2013). Cancer cell exosomes depend on cell-surface heparan sulfate proteoglycans for their internalization and functional activity. Proc Natl Acad Sci U S A.

[50] McKiernan J, Donovan MJ, O'Neill V, Bentink S, Noerholm M, Belzer S, Skog J, Kattan MW, Partin A, Andriole G, Brown G, Wei JT, Thompson IM (2016). A Novel Urine Exosome Gene Expression Assay to Predict High-grade Prostate Cancer at Initial Biopsy. JAMA Oncol.

[51] van Niel G, Bergam P, Di Cicco A, Hurbain I, Lo Cicero A, Dingli F, Palmulli R, Fort C, Potier MC, Schurgers LJ, Loew D, Levy D, Raposo G (2015). Apolipoprotein E Regulates Amyloid Formation within Endosomes of Pigment Cells. Cell Rep.

[52] Uhlén M, Fagerberg L, Hallström BM, Lindskog C, Oksvold P, Mardinoglu A, Sivertsson Å, Kampf C, Sjöstedt E, Asplund A, Olsson I, Edlund K, Lundberg E (2015). Proteomics. Tissue-based map of the human proteome. Science.

[53] McMahon GA, Petitclerc E, Stefansson S, Smith E, Wong MK, Westrick RJ, Ginsburg D, Brooks PC, Lawrence DA (2001). Plasminogen activator inhibitor-1 regulates tumor growth and angiogenesis. J Biol Chem.

[54] Fortenberry YM, Brandal SM, Carpentier G, Hemani M, Pathak AP (2016). Intracellular Expression of PAI-1 Specific Aptamers Alters Breast Cancer Cell Migration, Invasion and Angiogenesis. PLoS One.

[55] Hsu YC, Yuan S, Chen HY, Yu SL, Liu CH, Hsu PY, Wu G, Lin CH, Chang GC, Li KC, Yang PC (2009). A four-gene signature from NCI-60 cell line for survival prediction in non-small cell lung cancer. Clin Cancer Res.

[56] Langfelder P, Zhang B, Horvath S (2008). Defining clusters from a hierarchical cluster tree: the Dynamic Tree Cut package for R. Bioinformatics.

[57] Meckes DG (2014). Affinity purification combined with mass spectrometry to identify herpes simplex virus protein-protein interactions. Methods Mol Biol.

[58] Vizcaíno JA, Deutsch EW, Wang R, Csordas A, Reisinger F, Ríos D, Dianes JA, Sun Z, Farrah T, Bandeira N, Binz PA, Xenarios I, Eisenacher M (2014). ProteomeXchange provides globally coordinated proteomics data submission and dissemination. Nat Biotechnol.

[59] Liu H, Sadygov RG, Yates JR (2004). A model for random sampling and estimation of relative protein abundance in shotgun proteomics. Anal Chem.

[60] Old WM, Meyer-Arendt K, Aveline-Wolf L, Pierce KG, Mendoza A, Sevinsky JR, Resing KA, Ahn NG (2005). Comparison of label-free methods for quantifying human proteins by shotgun proteomics. Mol Cell Proteomics.

[61] McIlwain S, Mathews M, Bereman MS, Rubel EW, MacCoss MJ, Noble WS (2012). Estimating relative abundances of proteins from shotgun proteomics data. BMC Bioinformatics.

[62] Love MI, Huber W, Anders S (2014). Moderated estimation of fold change and dispersion for RNA-seq data with DESeq2. Genome Biol.

[63] Zhang L, Liu YZ, Zeng Y, Zhu W, Zhao YC, Zhang JG, Zhu JQ, He H, Shen H, Tian Q, Deng FY, Papasian CJ, Deng HW (2016). Network-based proteomic analysis for postmenopausal osteoporosis in Caucasian females. Proteomics.

[64] Kommadath A, Bao H, Arantes AS, Plastow GS, Tuggle CK, Bearson SM, Guan lL, Stothard P (2014). Gene co-expression network analysis identifies porcine genes associated with variation in Salmonella shedding. BMC Genomics.

[65] Langfelder P, Horvath S (2008). WGCNA: an R package for weighted correlation network analysis. BMC Bioinformatics.

[66] Dolédec S, Chessel D (1994). Co-inertia analysis: an alternative method for studying species-environment relationships. Freshwater Biology.

[67] Culhane AC, Perrière G, Higgins DG (2003). Cross-platform comparison and visualisation of gene expression data using co-inertia analysis. BMC Bioinformatics.

[68] Barrett T, Wilhite SE, Ledoux P, Evangelista C, Kim IF, Tomashevsky M, Marshall KA, Phillippy KH, Sherman PM, Holko M, Yefanov A, Lee H, Zhang N (2013). NCBI GEO: archive for functional genomics data sets--update. Nucleic Acids Res.

[69] Pfister TD, Reinhold WC, Agama K, Gupta S, Khin SA, Kinders RJ, Parchment RE, Tomaszewski JE, Doroshow JH, Pommier Y (2009). Topoisomerase I levels in the NCI-60 cancer cell line panel determined by validated ELISA and microarray analysis and correlation with indenoisoquinoline sensitivity. Mol Cancer Ther.

[70] Kohn KW, Zeeberg BM, Reinhold WC, Pommier Y (2014). Gene expression correlations in human cancer cell lines define molecular interaction networks for epithelial phenotype. PLoS One.

[71] Meng C, Kuster B, Culhane AC, Gholami AM (2014). A multivariate approach to the integration of multi-omics datasets. BMC Bioinformatics.

[72] Huang dW, Sherman BT, Lempicki RA (2009). Bioinformatics enrichment tools: paths toward the comprehensive functional analysis of large gene lists. Nucleic Acids Res.

[73] Huang dW, Sherman BT, Lempicki RA (2009). Systematic and integrative analysis of large gene lists using DAVID bioinformatics resources. Nat Protoc.

[74] Pathan M, Keerthikumar S, Ang CS, Gangoda L, Quek CY, Williamson NA, Mouradov D, Sieber OM, Simpson RJ, Salim A, Bacic A, Hill AF, Stroud DA (2015). FunRich: An open access standalone functional enrichment and interaction network analysis tool. Proteomics.

