# Supplementary material for: Proteomic profiling of NCI-60 extracellular vesicles uncovers common protein cargo and cancer type-specific biomarkers
Source: Oncotarget. 2016 Nov 24;7(52):86999–7015. doi: 10.18632/oncotarget.13569 (PMC5341331; doi:10.18632/oncotarget.13569)
Supplement: Supplementary file 1 [file oncotarget-07-86999-s001.pdf]

Proteomic profiling of NCI-60 extracellular vesicles uncovers common protein cargo and cancer type-specific biomarkers

SUPPLEMENTARY FIGURES AND TABLES

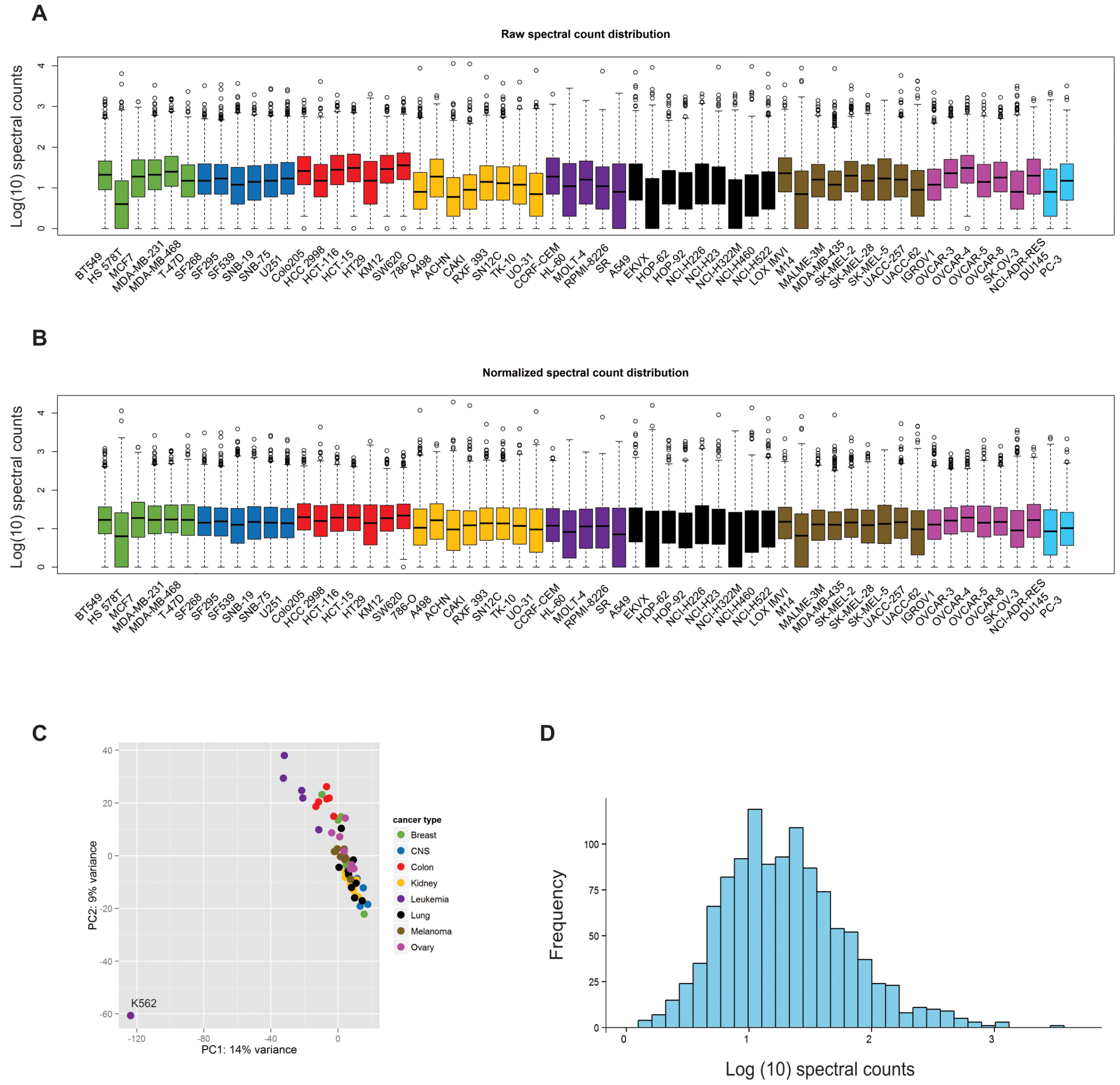

Supplementary Figure S1: Analysis of raw spectral count data. Related to Figure 3. Boxplots of **A.** raw spectral count distribution of EV isolates, and **B.** distributions following normalization by DESeq2. Boxes include the interquartile range (IQR). Maximum/minimum values extending beyond the first/third quartile ± 1.5 (IQR) are plotted as points. **C.** PCA plot of normalized data, revealing K562 as an outlying leukemia sample. **D.** Histogram of spectral count median log abundance for proteins identified in NCI-60 EV samples.

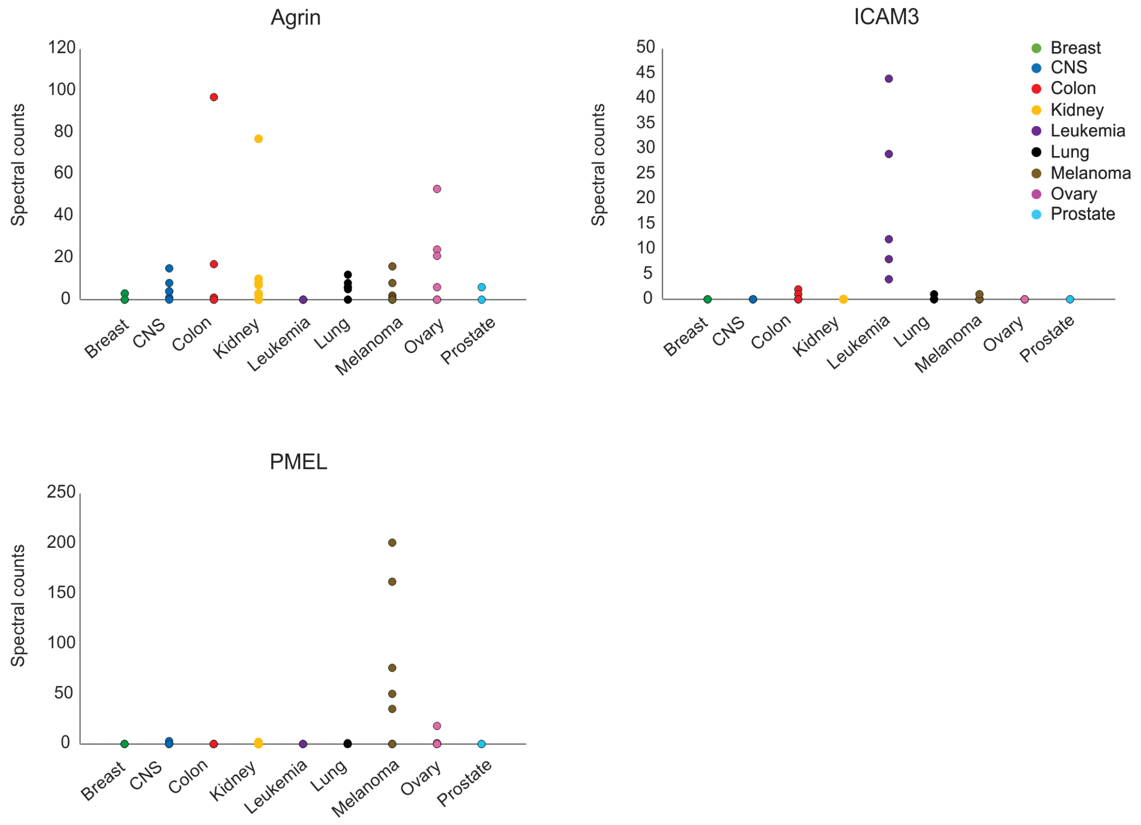

Supplementary Figure S2: Whole cell comparison of differentially expressed proteins. Related to Figure 3.

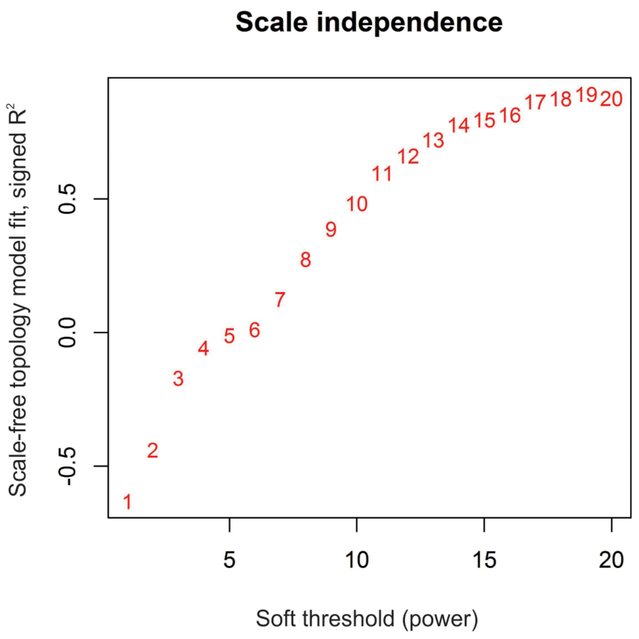

Supplementary Figure S3: Analysis of network topology for levels of soft-thresholding. Related to Figure 4.

Supplementary Table S1: MaxQuant output of all proteins identified in NCI-60 EV samples

See Supplementary File 1

Supplementary Table S2: Gene names associated with proteins found in the NCI-60, $[NCI\text{-}60]_{stringent}$, and Vesiclepedia databases

See Supplementary File 2

Supplementary Table S3: Spectral counts of 213 common proteins identified in all EV samples

See Supplementary File 3

Supplementary Table S4: Tissue-specific proteins

See Supplementary File 4

Supplementary Table S5: Proposed new EV markers

See Supplementary File 5

Supplementary Table S6: DESeq2 output of differentially-expressed proteins

See Supplementary File 6

Supplementary Table S7: Quantities of particles secreted per cell across the NCI-60

See Supplementary File 7

Supplementary Table S8: Output of WCGNA module protein membership

See Supplementary File 8

Supplementary Table S9: Comparison of core vesicle protein expression in whole cell and EV isolates

See Supplementary File 9
